# Luxation of the Jaw

**Published:** 1883-01

**Authors:** 


### Luxation of the Jaw.

The late Professor Gibson used to tell a good anecdote in regard to luxation of the jaw. An old and quite wealthy man came into the office of a surgeon, with a luxation of the jaw, and made motions to have it reduced. The jaw was reduced, and on being asked the fee the doctor mentioned an amount which the man regarded as entirely too much, and insisted on its being reduced one-half. The surgeon said no more about the fee, but began to talk, and pretty soon told a laughable story. The man began to laugh heartily, and out went the jaw. He again made signs to have it reduced, but the doctor said: "When you pay down my fee, I will put in your jaw."

